# Neurobiology and Therapeutic Potential of α5-GABA Type A Receptors

**DOI:** 10.3389/fnmol.2019.00179

**Published:** 2019-07-24

**Authors:** Tija C. Jacob

**Affiliations:** Department of Pharmacology and Chemical Biology, University of Pittsburgh School of Medicine, Pittsburgh, PA, United States

**Keywords:** GABA A receptor, alpha 5 subunit, autism, cognition, memory, development, negative and positive allosteric modulators

## Abstract

α5 subunit containing GABA type A receptors (GABA_A_Rs) have long been an enigmatic receptor subtype of interest due to their specific brain distribution, unusual surface localization and key role in synaptic plasticity, cognition and memory. These receptors are uniquely positioned to sculpt both the developing and mature hippocampal circuitry due to high overall expression and a distinct peak within the critical synapse formation period during the second postnatal week. Unlike the majority of other GABA_A_Rs, they exhibit both receptor clustering at extrasynaptic sites *via* interactions with the radixin scaffold as well as synaptic sites *via* gephyrin, thus contributing respectively to tonic currents and synaptic GABAergic neurotransmission. α5 GABA_A_R signaling can be altered in neurodevelopmental disorders including autism and mental retardation and by inflammation in CNS injury and disease. Due to the unique physiology and pharmacology of α5 GABA_A_Rs, drugs targeting these receptors are being developed and tested as treatments for neurodevelopmental disorders, depression, schizophrenia, and mild cognitive impairment. This review article focuses on advances in understanding how the α5 subunit contributes to GABA_A_R neurobiology. In particular, I discuss both recent insights and remaining knowledge gaps for the functional role of these receptors, pathologies associated with α5 GABA_A_R dysfunction, and the effects and potential therapeutic uses of α5 receptor subtype targeted drugs.

## Introduction

### Structure, Distribution and Composition

**Figure 1:**
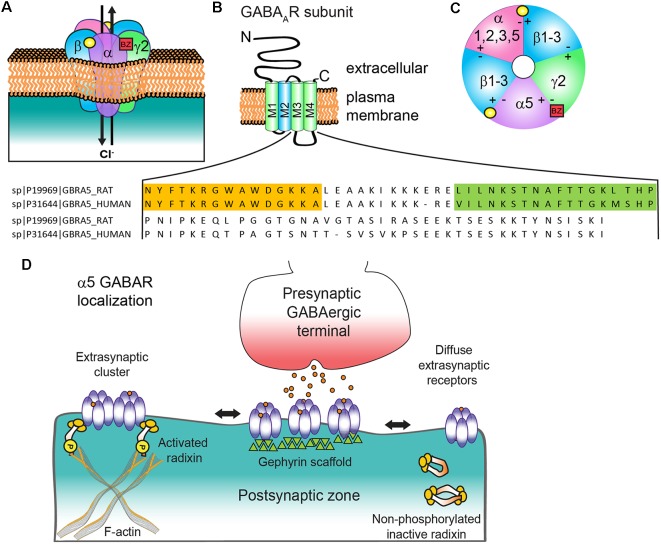
α_5_ subunit containing GABA type A receptor (α5 GABA_A_R) structure and subunit topology. **(A)** Generic synaptic GABA_A_R heteropentamer. Binding of the neurotransmitter GABA (yellow circle) at the αβ interface triggers ion channel opening and allows the rapid Cl^−^ influx and membrane hyperpolarization. Benzodiazepines (BZ, red box) bind at the interface of an α1/2/3/5 and γ2 subunit. **(B)** All subunits have a common topology including an extracellular N-terminal domain (NT), short C-terminal tail (CT), and four transmembrane regions (M1–4) which compose the transmembrane domain. M2 (blue) contributes to formation of the receptor ion channel pore, while the large cytoplasmic domain between M3 and M4 (CD) contains sites for protein interactions and post translational modifications that modulate channel function and/or trafficking: amino acid residue alignment of rat and human α5 CD with radixin binding domain (orange highlighted residues, from Loebrich et al., 2006) and gephyrin interacting region (green highlighted residues, from Brady and Jacob, 2015). **(C)** α5 GABA_A_R extracellular representation with potential subunit combinations. **(D)** Schematic of α5 GABA_A_R clustering mechanisms at extrasynaptic and synaptic locations with radixin and gephyrin. Phosphorylated radixin interacts with receptors and actin, while with dephosphorylation radixin N-terminal FERM and C-terminal F-actin binding domains interact and form inactive monomers or dimers.

GABA type A receptors (GABA_A_Rs) are heteropentameric ligand-gated chloride (Cl^−^) ion channels typically composed of two α (α1–6), two β (β1–3), and one γ (γ1–3) or δ subunit (Figure 1A). The common structure of individual subunits consists of a large extracellular N-terminus (NT), four transmembrane α-helices (M1–4) and a barely extruding extracellular C-terminus (CT). The conserved hydrophobic M domains are connected by small regions with a larger cytoplasmic domain between M3 and M4 (CD) that mediates interactions with intracellular proteins critical for receptor trafficking and surface localization (Figure 1B). Receptors can contain two different α or β subunits that are arranged in a counterclockwise configuration of γ-β-α-β-α (Figure 1C). The two αβ NT interfaces form GABA binding sites composed of the principal (+) side of the β subunit and the complementary α subunit (−) side, while a single α+(1, 2, 3 or 5)/γ2- interface generates the primary binding site for benzodiazepines, which are allosteric positive modulators of the GABA_A_R and an important clinical sedative-hypnotic-anxiolytic drug class. Several recent high resolution cryo-electron microscopy studies have provided unprecedented structural information for GABA_A_R (Phulera et al., 2018; Zhu et al., 2018; Laverty et al., 2019; Masiulis et al., 2019), advancing understanding of receptor architecture, principles of assembly, and binding of various ligands: GABA, bicuculline (antagonist), picrotoxin (channel blocker), and benzodiazepines. The channel properties, subcellular localization and pharmacological sensitivity of a GABA_A_R are defined by the subunit composition. While α5 containing GABA_A_Rs makeup only approximately 5% of the total receptor population in the brain, they are highly expressed in both the hippocampus and olfactory bulb. They represent close to 25% of all hippocampal GABA_A_R (Olsen and Sieghart, 2009) and are particularly abundant in CA1 and CA3. In the olfactory bulb, over a third of the neurons in the internal granule cell layer have α5 GABA_A_Rs (Sur et al., 1999), although the function here is unknown. α5 GABA_A_Rs are also expressed in the spinal cord, where they contribute to presynaptic inhibitory control over sensory-motor transmission (Lucas-Osma et al., 2018) and are also implicated in resolution of hyperalgesia (Perez-Sanchez et al., 2017). Other brain regions where these receptors are found at lower levels include the cortex, subiculum, hypothalamus, sympathetic preganglionic neurons, and amygdala (Martin et al., 2009a).

Early pharmacological analysis indicated rat and human hippocampal α5 GABA_A_Rs have α5β3γ2 characteristics (Sur et al., 1998). However, sequential immunoprecipitation from hippocampal tissue identified that α1/α5 heteromers constitute approximately 9% of the α1 GABA_A_Rs and α2/α5 heteromers constitute about 20% of the α2 population in the hippocampus (Araujo et al., 1999; del Río et al., 2001). More recent mass spectrometry analysis of affinity purified α5 GABA_A_Rs from mouse hippocampus supported association of α5 with α1–3, β1–3 and both γ2S and γ2L isoforms (Ju et al., 2009). A recent comparison of α5β1–3γ2L GABA_A_Rs in HEK cells co-cultured with neurons revealed robust inhibitory postsynaptic currents (IPSCs) with slow decay rates and isoform-specific effects of pharmacological inhibitors (Chen et al., 2017). Importantly, in mixed alpha subunit GABA_A_Rs there appears to be preferential assembly of α5 and γ2 together, generating a benzodiazepine binding site with α5 subunit pharmacology (Araujo et al., 1999; del Río et al., 2001). Thus for a mixed α5 GABA_A_R, the other alpha subunit is essentially pharmacologically inactive for benzodiazepines and other alpha/gamma subunit interface binding drugs (i.e., the “Z-drugs” for insomnia treatment zolpidem, zopiclone, zaleplon). Mutation of the α5 subunit H105 residue, a key alpha subunit residue required for forming the benzodiazepine binding site with the γ2 subunit, led to repositioning of α5 H105R subunits into the pharmacologically inactive alpha subunit location (Balic et al., 2009). Interestingly, our recent mass spectrometry analysis identified a specific increase in α5βγ2 containing receptors in the cortex following diazepam injection, consistent with benzodiazepine exposure leading to modification of GABA_A_R composition and potentially drug effects through α5 plasticity (Lorenz-Guertin et al., 2019).

## Cellular and Circuit Localization

### Subcellular Localization

Controversies regarding α5 GABA_A_R subcellular localization in the literature have mirrored debates about its functional impact on GABAergic neurotransmission. Due to their initial identification as a key generator of hippocampal tonic current (Caraiscos et al., 2004; Glykys and Mody, 2006; Bonin et al., 2007), α5 GABA_A_Rs were generally considered extrasynaptic receptors, despite earlier evidence for synaptic clustering on dendrites and the axon initial segment (Brünig et al., 2002; Christie and de Blas, 2002; Serwanski et al., 2006). α5 GABA_A_Rs predominantly mediate tonic inhibition in hippocampal CA3 and CA1 pyramidal neurons, cortical neurons (layer 5) and are contributors to tonic inhibition in dentate gyrus granule cells (Glykys et al., 2008; Herd et al., 2008). Immunocytochemistry indicates an extensive extrasynaptic presence of α5 GABA_A_Rs (Brünig et al., 2002; Crestani et al., 2002). However, this receptor subtype is unique in displaying surface clustering at extrasynaptic locations rather than a uniformly diffuse extrasynaptic distribution. Regions within the large cytoplasmic domain between M3 and M4 regulate subcellular clustering of α5 GABA_A_Rs *via* interactions with radixin and gephyrin scaffolds (Figure 1D). Extrasynaptic clustering is mediated by radixin, an ezrin/radixin/moesin (ERM) family member that links actin to the plasma membrane (Loebrich et al., 2006). Phosphorylated radixin scaffolds α5βγ2 receptors to the actin cytoskeleton, ultimately reducing diffusion rates and concentrating channel activity away from axon terminals (Hausrat et al., 2015). Treatment with GABA promotes radixin phosphorylation and retention of α5 GABA_A_Rs extrasynaptically, while AMPA, a ligand for ionotropic glutamatergic GluA type receptors, leads to dephosphorylation, an increase in synaptic α5-subunit receptors and an increase in slowly decaying miniature IPSCs (mIPSCs). Further support for the specific contribution of α5 GABA_A_Rs to slowly decaying IPSCs is seen in early neurodevelopment during the switch from α5 to α1 and α3 subunit expression (Pangratz-Fuehrer et al., 2016). Important areas of further investigation include assessment of the level and role of α5 GABA_A_Rs associated with radixin or gephyrin in the developing and adult brain and plasticity mechanisms regulating these interactions.

Functional studies indicate the α5 subunit is also important for phasic events including: spontaneous inhibitory postsynaptic currents (sIPSCs), evoked IPSCs (eIPSCs) and GABA_slow_ IPSCs (Collinson et al., 2002; Prenosil et al., 2006; Zarnowska et al., 2009; Vargas-Caballero et al., 2010). Consistent with a synaptic role for α5 GABA_A_Rs, we demonstrated that the α5 subunit directly interacts with the gephyrin synaptic scaffold, with approximately half of surface α5 GABA_A_Rs being synaptically localized throughout the first 3 weeks of circuit development (Brady and Jacob, 2015). Single particle tracking studies measured reduced diffusion of surface α5 GABA_A_Rs at synapses (Renner et al., 2012) and similar to other synaptic receptors, α5 GABA_A_Rs showed an increase in diffusion with negative modulator DMCM treatment (Lévi et al., 2015). Further studies are needed to determine both acute and prolonged effects of α5 preferring GABA_A_R drugs on receptor diffusive properties and surface stability.

### Cell Type and Input-Specific Expression

α5 GABA_A_Rs show input-specific synaptic localization and function in different brain regions both for pyramidal cells and interneurons. Recent work demonstrates preferential localization of α5 GABA_A_Rs to inhibitory synapses on dendrites of somatostatin-expressing interneurons in CA1 that are targeted by vasoactive intestinal peptide and calretinin-positive interneurons (Magnin et al., 2019). Somatostatin interneurons and NO-synthase-positive neurogliaform cells target α5 GABA_A_Rs on dendrites of hippocampal CA1 pyramidal neurons to generate slow IPSCs (Schulz et al., 2018). Importantly, these outward-rectifying α5-GABA_A_Rs generate a greater hyperpolarizing current at slightly depolarized membrane potentials, thereby having a large impact on NMDA-receptor-activation and action potential firing in pyramidal neurons. In the cortex, pyramidal cells exhibit dendritically localized α5 GABA_A_Rs at sites innervated by bitufted interneurons (an SST positive neuron class; Ali and Thomson, 2008). A recent human and mouse prefrontal cortex gene expression study determined that the majority of α5 GABA_A_Rs are in pyramidal cells, followed by parvalbumin interneurons (Hu et al., 2018). Interestingly, α5 GABA_A_R mRNA was uniquely expressed in human SST interneurons, albeit at a low level. As deficits in both GABAergic signaling and SST signaling (Fuchs et al., 2017) have been identified as contributors to major depressive disorder, this data suggests positive modulation of α5 GABA_A_R could be therapeutic by multiple mechanisms. It is clear that improving understanding of GABA_A_R subtype subcellular (extrasynaptic vs. synaptic) and circuit-specific localization and function are critical areas of current research and future pharmacological development (reviewed in Engin et al., 2018).

## Functional Role of α5 GABA_A_Rs

### Neuronal Excitability, Learning and Memory

Genetic and pharmacological studies in rodents demonstrate that α5 GABA_A_Rs are key in learning and memory processes (reviewed in Martin et al., 2009a). The two primary mouse models used in studying the α5 GABA_A_R contribution to cognitive processes are the α5 subunit knockout mice (*Gabra5^−/−^*) and the α5H105R point mutation mice. Although originally generated to render α5 receptors insensitive to benzodiazepines, α5H105R mice also have a 25% decrease in hippocampal α5 protein level (Crestani et al., 2002). As described earlier, *Gabra5*^−/−^ mice showed a reduction in diverse types of phasic GABA_A_R currents and the tonic current. Behaviorally, the increased excitability of *Gabra5*^−/−^ hippocampal pyramidal neurons was correlated with improved performance in a spatial learning behavior (Collinson et al., 2002), though later studies were not able to replicate this result (Cheng et al., 2006; Martin et al., 2009b). However, both *Gabra5*^−/−^ and α5H105R mice show enhanced trace fear conditioning, a hippocampal learning task, while performing similarly to wild-type mice in a cued fear conditioning assay, which relies on the amygdala, hippocampus, and cortex (Crestani et al., 2002; Martin et al., 2009b). Long-term potentiation (LTP), the cellular correlate of learning and memory, is constrained by GABA_A_R-mediated inhibition. *Gabra5*^−/−^ mice showed a reduced threshold for LTP induction with 10–20 Hz stimulation (Martin et al., 2010). In addition, *Gabra5*^−/−^ mice showed greater power of kainate-induced gamma frequency oscillations (Towers et al., 2004), and knockout of delta and α5 subunits led to spontaneous gamma oscillations in CA3 (Glykys et al., 2008). Gamma oscillations occur in a range of cognitive states including memory processing, are thought to support neural coding of environmental information and are disturbed in some psychiatric disorders (reviewed in Lisman and Buzsáki, 2008). In summary, a reduction in α5 inhibition may improve learning and memory through enhanced neuronal firing and network oscillatory activity.

### Development

In contrast to their inhibitory role in the mature nervous system, GABA_A_Rs can promote excitation in newly forming circuits, allowing chloride efflux to produce membrane depolarization which promotes calcium entry, dendritic outgrowth, synaptogenesis and unsilencing of glutamatergic synapses (reviewed in Ben-Ari et al., 2007). α5 GABA_A_Rs are particularly well positioned to sculpt early hippocampal circuit development due to exceptionally high expression that peaks in the first two postnatal weeks (Liu et al., 1998; Ramos et al., 2004; Yu et al., 2014; Bader et al., 2017), and receptor localization at both extrasynaptic and synaptic sites. During the first postnatal week, tonic α5 currents enhance cell excitability and synaptic activity, facilitating the induction of giant depolarizing potentials, which are important for early network maturation (Ben-Ari, 2002; Marchionni et al., 2007). Importantly, GABAergic activation of circuit formation also occurs with newborn neurons integrating into networks in the adult mammalian brain *in vivo* (Ge et al., 2006). A few *in vitro* pharmacological and genetic studies have supported the role of α5 GABA_A_Rs in dendritic development. Cultured hippocampal neurons treated with an α5-specific negative allosteric modulator (NAM; RY-80) exhibited decreased dendritic arborization and reduced expression of the AMPA type glutamate receptor GluA2 subunit (Giusi et al., 2009). To investigate the role of α5 GABA_A_Rs in emerging circuits, we genetically manipulated α5 binding to gephyrin, increasing or decreasing the ratio of extrasynaptic/synaptic α5 GABA_A_Rs (Brady and Jacob, 2015). Interestingly, reducing synaptic α5 GABA_A_Rs promoted dendritic outgrowth at the expense of dendritic spine maturation in hippocampal neurons. Consistent with these findings, recent work showed that single-cell deletion of *Gabra5* in adult-born dentate gyrus granule cells caused severe alterations of migration and dendrite development (Deprez et al., 2016). Further research is needed to elucidate the specific role of the α5 subunit in dendritic architecture, both during development and in adult neurogenesis.

### Genetic Disorders with Altered α5 GABA_A_R Neurotransmission

While acute reduction in α5 GABA_A_Rs has shown potential for improving cognition and memory, further studies both in mouse models and human patients link long term reduction with significant pathologies. Reduced α5 GABA_A_R levels, function or protein interactions have been observed in patients with neurodevelopmental disorders including intellectual disability, epilepsy and autism. Common conditions among these disorders include cognitive impairments, increased anxiety, autism-related behaviors, sleep disorders and epilepsy susceptibility. Analogous behavioral changes and pathologies are observed in mouse models including *Gabra5^−/−^*mice (Zurek et al., 2016; Mesbah-Oskui et al., 2017), Fragile X syndrome model mice (*Fmr1*^−/−^ mice, Bakker and Oostra, 2003), and other mouse models of ASD (reviewed in Kazdoba et al., 2016). *Fmr1*^−/−^ mice show downregulation of α5 GABA_A_R and a deficit in tonic inhibition (Curia et al., 2009). Subsequent studies of α5H105R mice identified behavioral changes including hyperactivity and impaired encoding of object location memories (Hauser et al., 2005; Prut et al., 2010), although some behavioral changes may be attributed to subunit ordering rearrangements in a mixed alpha subunit GABA_A_R (see earlier, Composition).

The most commonly reported loci of chromosomal abnormalities in ASD patients are found in the q11.2–13 region on chromosome 15 (Hogart et al., 2010). Among the genes in this region are the α5, β3, and γ3 subunits. An autism patient exome study identified mutations including α5G113A (NT), α5V204I (NT) and mutations in the extrasynaptic anchor radixin: T516I, P471T, D197H, A496V (Zurek et al., 2016). Exome sequencing of sporadic genetic epilepsy patients identified α5V204I (NT), α5W280R (M1), α5S402A (CD) and α5P453L (CT) mutations (Hernandez et al., 2016). Recombinant studies of these mutant α5β3γ2 GABA_A_Rs indicated no pronounced changes in surface or total α5 levels, while functional deficiencies ranged from reduced currents and gating defects to altered channel activation and deactivation. A V294L (M2, pore-lining helix) mutation identified in a patient with severe early-onset epilepsy and developmental delay showed receptors with 10 times greater GABA sensitivity, although maximal GABA currents were reduced by increased receptor desensitization (Butler et al., 2018). An autism patient pilot PET imaging study with the α5 preferring tracer [11C]Ro15-4513 identified reduced α5 binding across multiple brain regions (Mendez et al., 2013), while another recent study showed changes in a GABA-sensitive perceptual task without differences in binding (Horder et al., 2018). As both studies were without genetic information, this suggests further testing with patient stratification by exome data could provide greater insight. Despite being a genetically heterogeneous disorder, the potential utility for mechanism-based GABA_A_R pharmacologic treatment with ASDs is supported by shared pathologies both in patients and related mouse models.

## α5 GABA_A_R Therapeutics

NAMs that selectively reduce α5 GABA_A_R function have been heavily pursued for the potential development of cognitive enhancing or “smart” drugs. The following are a selection of α5 GABA_A_R NAMs: L-655,708, α5IA, Ro15-4513, MRK-016, RO4938581, and RY-80 (reviewed in Clayton et al., 2015; Sieghart and Savic, 2018). Importantly, α5 NAMs did not exhibit the convulsant or pro-convulsant activity of more general alpha subunit NAMs, had good oral bioavailability and easily crossed the blood brain barrier (reviewed in Atack, 2011). In contrast to NAMs which act *via* the GABA_A_R benzodiazepine binding site, S44819 was recently identified as a competitive antagonist of GABA at α5 GABA_A_R and showed similar pro-cognitive effects as NAMs: blocking α5-GABA_A_R tonic current, enhancing LTP, reversing scopolamine-induced impairment of spatial working memory and enhancing object recognition memory (Ling et al., 2015; Etherington et al., 2017). Finally, recent evidence for beneficial effects of positive allosteric modulators (PAMs) in aged brain cognition, autism, depression and schizophrenia has bolstered α5 PAM drug development. A selection of α5 preferring PAMs includes SH-053-R-CH3-2′F, MP-III-022, and GL-II-73 (Sieghart and Savic, 2018; Prevot et al., 2019). Potential therapeutic applications for α5 preferring NAMs and PAMs are discussed below with a focus on CNS specific uses (Table 1), although important remaining questions exist for both *in vivo* specificity and receptor subtype selectivity as recently reviewed (Sieghart and Savic, 2018).

**Table 1:** Summary table of α_5_ subunit containing GABA type A receptor (α5 GABA_A_R) targeted drugs and potential utility.

Drug type	Reduce α5 GABA_A_R activity (NAM or competetive antagonist)	Increase α5 GABA_A_R activity (PAM)
Compound	L-655, 708, α5IA, Ro15-4513, MRK-016, RO4938581, RY-80, S44819 (competetive antagonist)	SH-053-R-CH3-2′F, MP-III-022, Compound 44, GL-II-73
Therapeutic potential	Procognition/smart drugs	Mild cognitive impairment in aging
	Neurodevelopmental disorders with excessive GABAergic neurotransmission	Neurodevelopmental disorders with insufficient inhibitory tone
	Inflammation induced mild cognitive impairment	Depression
	Post-anesthesia memory blockade	Schizophrenia

*This includes drugs that can reduce α5 GABA_A_R activity [negative allosteric modulators (NAMs) and the competitive antagonist S44819] and positive allosteric modulators (PAMs) that enhance α5 GABA_A_R activity. Representative compounds and therapeutic potential are listed*.

### NAM α5 GABA_A_R Therapeutic Applications

#### Pro-cognition

The ability of α5 preferring NAMs to enhance learning and memory in rodents provided crucial evidence for the importance of α5 GABA_A_Rs in these processes (Chambers et al., 2002, 2003; Street et al., 2004). The α5 NAM L-655,708, which shows approximately 50–100-fold selectivity for α5 GABA_A_Rs, reduced tonic inhibition, enhanced LTP, improved performance in the Morris water maze and generated spontaneous gamma oscillations in the CA3 region of the hippocampus (Caraiscos et al., 2004; Atack et al., 2006; Glykys et al., 2008). However anxiogenic activity and pharmacokinetics (reviewed in Atack, 2011) prevented its use in humans. Although α5IA was non-anxiogenic and reduced ethanol-induced learning impairment in young volunteers, prolonged use was prevented by high dose renal toxicity (Atack, 2010). MRK-016 showed pro-cognitive efficacy and was non-anxiogenic; poor compound tolerance in the elderly stopped further clinical development (Atack et al., 2009). Efforts to develop clinically successful α5 NAM are ongoing.

#### Developmental Disorders

Down syndrome mice (Ts65Dn) show cognitive impairment due to excessive GABAergic inhibition. Acute treatment with α5IA reversed deficits in novel object recognition and spatial learning and was able to restore deficits of immediate early genes expression during memory processing (Braudeau et al., 2011). Although Ts65Dn mice show no major changes in α5 GABA_A_R levels (Deidda et al., 2015), growing evidence indicates increased α5 GABA_A_R activity is an important pathological component, as genetic ablation of α5 GABA_A_Rs partially rescues learning, LTP and neuromorphological changes (Vidal et al., 2018). Furthermore, a recent study revealed a specific increase in GABA_A_R dendritic inhibition in Ts65Dn mice that led to reduced NMDAR activation and impaired LTP that could be restored with α5 NAM treatment (Schulz et al., 2019). *Rdx^−/−^*mice have increased GABAergic inhibition *via* enhanced α5 synaptic levels, impaired short-term memory and a reversal learning deficit, with the latter being improved with α5IA treatment (Hausrat et al., 2015). The subsequently identified α5 NAM RO4938581, with high affinity and efficacy at α5 GABA_A_Rs vs. α1–3 GABA_A_Rs (Ballard et al., 2009), demonstrated efficacy in Ts65Dn mice at improving spatial memory, reversing LTP deficits, and restoring neurogenesis while reducing both hyperactivity and the enhanced density of hippocampal GABAergic boutons (Martínez-Cué et al., 2013). Although these pharmacological successes led to a Phase II clinical trial for a related compound RG1662 (Hoffman-La Roche) in Down syndrome patients, the trial did not meet the primary and secondary endpoints of improved cognition and function.

#### Inflammation Induced Mild Cognitive Impairment and Post Anesthesia Memory Blockade

Increased systemic inflammation caused by pathological events such as stroke, infection, and traumatic brain injury is associated with memory problems during recovery from the initial insult. In an acute inflammation model, increased tonic α5 GABA_A_R current and surface levels *via* P38 MAPK signaling was central to generating inflammation induced memory deficits (Wang et al., 2012). Importantly, these inflammation induced memory impairments were absent in *Gabra5*^−/−^ mice and could be blocked by treatment with the α5 NAMs L-655,708 or MRK-016. Similarly, following stroke injury, tonic inhibition is increased in the peri-infarct zone, and L-655,708 treatment from 3-days post-stroke increases functional recovery (Clarkson et al., 2010). Gabra5^−/−^ mice also exhibited improved motor recovery post-stroke. Sustained upregulation of α5 GABA_A_Rs is also indicated in memory blockade following anesthesia (Zurek et al., 2014). Both the injectable anesthetic etomidate and the inhaled anesthetic isoflurane increase α5 GABA_A_R tonic conductance, promoting the amnesic properties of these drugs (Cheng et al., 2006; Martin et al., 2009b; Saab et al., 2010). Pharmacological inhibition of α5 GABA_A_Rs reduces anesthetic potentiation of GABA_A_Rs (Lecker et al., 2013) and restores recognition memory in mice after anesthesia. Recent investigation of age-dependent efficacy of L-655,708 showed that α5 NAM treatment prior or following anesthesia restored spatial learning and memory in young rats, while aged rats only showed improvement with α5 NAM treatment prior to anesthesia (Zhao et al., 2019). Importantly, low dose isoflurane downregulated α5 mRNA in aging hippocampal neurons but upregulated α5 mRNA in neurons from young animals. This suggests different approaches will be needed to improve post anesthesia memory blockade in young vs. aged populations.

### PAM α5 GABA_A_R Therapeutic Applications

#### Neurodevelopmental Disorders

Mouse models of neurodevelopmental disorders that present with insufficient inhibitory tone show improvement with positive modulators of GABA_A_R signaling. In the Scn1a+/− mouse model of Dravet syndrome, a severe childhood epileptic encephalopathy syndrome with hyperactivity and autism behaviors, abnormal social behaviors and fear memory deficits were rescued following treatment with a benzodiazepine, clonazepam (Han et al., 2014). In an ASD mouse model with reduced GABA_A_R-mediated inhibition, the BTBR T+tf/J mouse, the α2,3 and 5 PAM L-838,417, improved deficits in social interaction, repetitive behaviors, and spatial learning (Han et al., 2014).

#### Mild Cognitive Impairment in Aging

Although α5 GABA_A_R NAMs enhance memory in young rodents, it appears positive modulation may be more therapeutic in aging brains impaired by excess activity. Particularly in disorders such as Alzheimer’s which are hallmarked by overexcitation (Ambrad Giovannetti and Fuhrmann, 2019), enhanced cognition may be achieved with reducing pathological excitability, as observed with the FDA approved NMDAR antagonist memantine. Furthermore, there is growing evidence for a general decline in GABAergic inhibitory tone in aging humans, monkeys and rodents (Rozycka and Liguz-Lecznar, 2017; Lissemore et al., 2018). From this newer perspective, an α5 GABA_A_R PAM focused approach (Compound 44) identified improved hippocampal-dependent memory in aged rats with cognitive impairment (Koh et al., 2013).

#### Depression and Schizophrenia

Another important unmet need where α5 GABA_A_Rs PAM pharmacotherapy may be applicable is in the development of new fast-acting anti-depressant drugs. Most current antidepressants act on the monoaminergic systems, and are only moderately therapeutically efficacious after dosing for several weeks. Significant evidence links GABAergic deficits with major depressive disorders (MDD) (Luscher et al., 2011). Investigation of anti-depressant activity of the α5 PAM SH-053-2′F-R-CH3 showed stress reduction in female mice both as an acute and chronic treatment (Piantadosi et al., 2016). Although male mice did not respond to PAM treatment, they also failed to show the upregulation of Gabra5 gene expression following unpredictable chronic mild stress seen in female mice. This particular PAM was also able to reverse pathological increases in dopaminergic activity in the MAM-model of schizophrenia (Gill et al., 2011). GL-II-73 a recently developed α5 preferring PAM showed anxiolytic and antidepressant efficacy, reversing stress-induced and age-related working memory deficits both in male and female mice (Prevot et al., 2019). Somewhat contradictory to this data and the GABA deficit hypothesis of MDD, α5 NAM have also shown rapid antidepressant actions in mice, potentially via ketamine like mechanisms of disinhibition (Fischell et al., 2015; Zanos et al., 2017).

## Conclusion

Due to the unique physiology and pharmacology of α5 GABA_A_Rs, these receptors are being targeted and tested as treatments for neurodevelopmental disorders, mild cognitive impairment, depression and schizophrenia. The recent cryo-EM studies of heteropentameric synaptic GABA_A_Rs and binding of GABA, antagonists, and benzodiazepines should further advance α5 subtype specific structure-based drug design. Despite the progress in understanding of α5 GABA_A_R neurobiology, comparatively little is understood regarding mechanisms that regulate α5 GABA_A_R trafficking, stability, and both synaptic and extrasynaptic clustering. Furthermore, understanding of α5 GABA_A_R plasticity occurring from endogenous signaling mechanisms and from drug treatments in the developing, mature and aging brain will be needed to effectively and safely advance therapeutic application of α5 GABA_A_R preferring drugs.

## Author Contributions

TJ prepared the figure, table and wrote the manuscript.

## Conflict of Interest Statement

The author declares that the research was conducted in the absence of any commercial or financial relationships that could be construed as a potential conflict of interest.
